# Editorial: Emotion and Behavior

**DOI:** 10.3389/fpsyg.2016.00313

**Published:** 2016-03-07

**Authors:** Fritz Strack, Paul Pauli, Peter Weyers

**Affiliations:** Department of Psychology, University of WürzburgWürzburg, Germany

**Keywords:** emotions, behavior, approach, avoidance, impulsive behavior

Within the field of psychology, there exists a strict division between disciplines that focus on “normal” vs. “dysfunctional” behavior. Despite its practical value, this distinction may be an obstacle that prevents cross-fertilization. While seemingly dysfunctional mechanisms have long been used to understand the regularities of human perception and cognition, behavioral aberrations have rarely been employed to explain the normality of human action.

The current collection of research reports is meant to overcome this obstacle in the domain of emotion. In particular, the contributors to this special issue share the conviction, that human behavior is not solely determined by the reflective evaluation of its anticipated consequences. Instead, affective impulses are also relevant in fueling approach or avoidance. This is particularly obvious when it comes to phenomena of temptation or addiction when impulsive mechanisms gain the upper hand. In a related fashion, a phobic person typically *knows* that a phobic situation may not be dangerous although he *feels* afraid and acts accordingly.

These observations, along with a great number of experimental results, suggest that emotion and behavior may be linked in at least two ways that can be described as *reflective* and *impulsive*. In a more systematic fashion, the two psychological mechanisms have been described in the context of the Reflective-Impulsive Model (RIM; Strack and Deutsch, 2004, 2015), which provides a conceptual orientation for the reported research.

A second denominator of the collected papers is the attempt to merge different levels of analysis. Specifically, they range from basic neuronal to social approaches with the aim to understand the different ways of interaction between emotion and behavior.

The basic themes to which the selected papers contribute are: (a) basic aspects of emotional-impulsive processing, (b) emotional processes underlying approach, and (c) emotional processes underlying avoidance.

**Basic aspects of emotional-impulsive processing** are addressed by *Kozlik, Neumann, and Lozo* who summarize findings suggesting that mechanisms of evaluative coding may better account for approach-avoidance behaviors than the principles of motivational orientation. *Seibt, Mühlberger, Likowski, and Weyers* address the phenomenon of facial mimicry in social situations, i.e., in response to emotional facial expressions of others. Based on results from EMG and fMRI, the authors identify the basic psychological processes that explain congruent and incongruent facial responses. Findings on multimodal interactions between pictures and sounds in their impact on emotional experiences are reviewed by *Gerdes, Wieser, and Alpers*. They conclude on basic interaction mechanisms leading to a congruent emotional experience.

***Emotional processes underlying approach*** *are discussed by Seibt, Häfner, and Deutsch* with regard to sexual approach as a function of gender and both objective and subjective deprivation. Their results demonstrate greater approach for men than for women and show that subjective desire mediates objective deprivation as a determinant. *Wu, Winkler, Wieser, Andreatta, Li, and Pauli* focus on the approach motivation related to craving in smokers. They report that heavy smokers—against common assumptions—are capable to regulate emotion via deliberate reappraisal and suggest that such emotion regulation strategies might be used in treatment to learn to regulate craving too. *Deutsch, Smith, Kordts-Freudinger, and Reichardt* focus on relief, which is assumed to elicit approach behavior, and propose an integrative relief model that links affect, emotion, and motivational systems.

***Emotional processes underlying avoidance*** *are* reviewed by *Diemer, Alpers, Peperkorn, Shiban, and Mühlberger* with a focus on the domain of virtual reality as a means to study the impact of perception and presence on emotional reactions, here fear and anxiety. *Wieser, Gerdes, Reicherts, and Pauli* present results on how pain and the resulting avoidance motivation influence the processing of emotional expressions and show an asymmetry in its impact on the processing of pleasant and unpleasant faces. The emotion of surprise is the topic of the contribution by *Topolinski and Strack*. Their work focusses on the accompanying facial activities and the role of negative affect.

Most of the contributing authors collaborated during their time at the University of Würzburg, Germany, as members of the research group “Emotion and Behavior” funded by the German Research Foundation (DFG, FOR 605). The editors thank all contributors for excellent collaborations during the last years.

## Author contributions

All authors listed, have made substantial, direct and intellectual contribution to the work, and approved it for publication.

## Funding

Funded by DFG FOR-605.

### Conflict of interest statement

The authors declare that the research was conducted in the absence of any commercial or financial relationships that could be construed as a potential conflict of interest.
